# Supplementary material for: Sulfide-Responsive Transcription Control in Escherichia coli
Source: Microorganisms. 2025 Feb 5;13(2):344. doi: 10.3390/microorganisms13020344 (PMC11858517; doi:10.3390/microorganisms13020344)
Supplement: Supplementary file 1 [file microorganisms-13-00344-s001.zip › Supp_info/Sup_Fig.pdf]

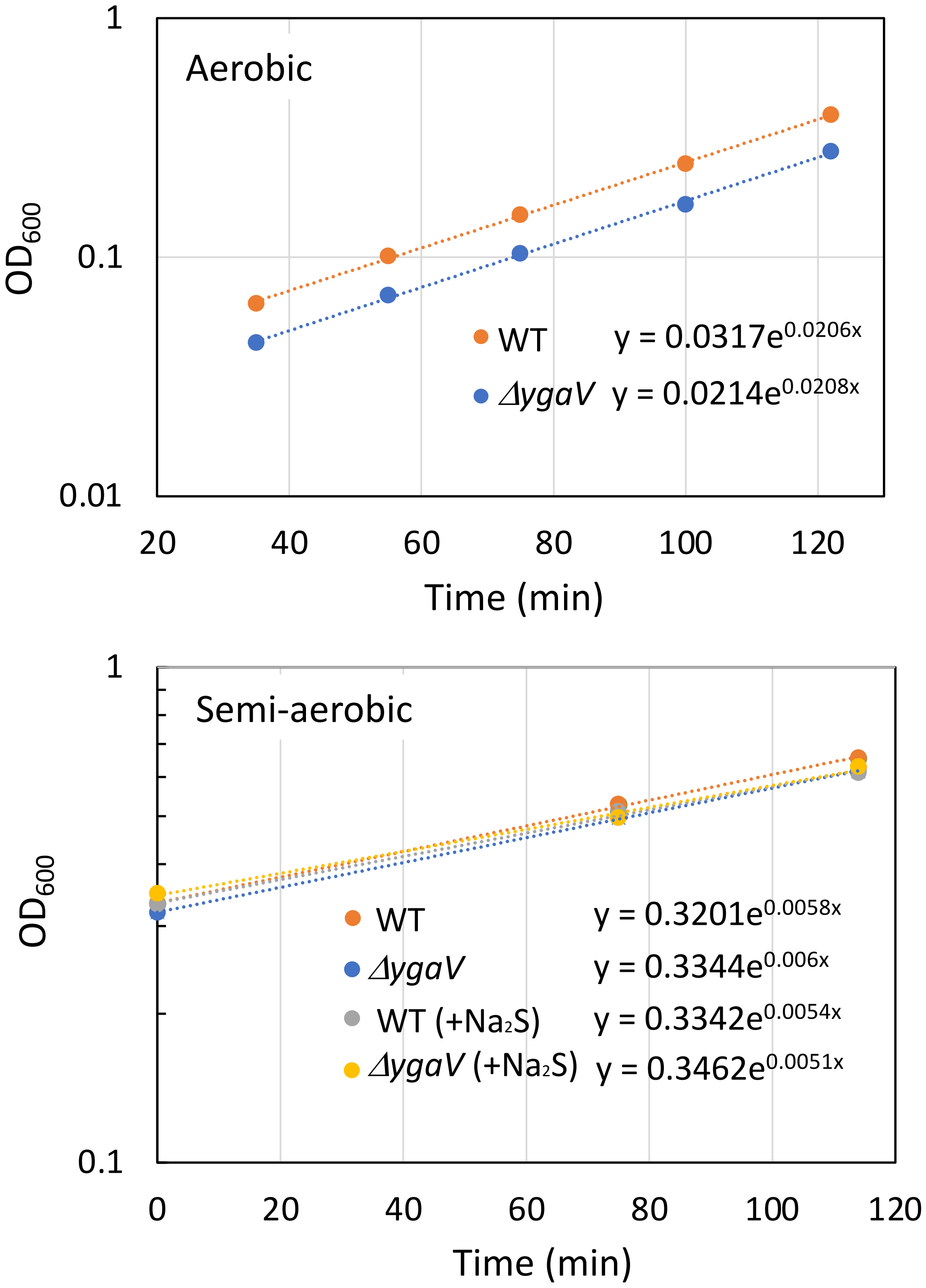

Supplemental Figure S1 Growth curves of WT and *ΔygaV* mutant grown under aerobic and semi-aerobic conditions with (+Na_2S) or without 0.2 mM Na_2S.

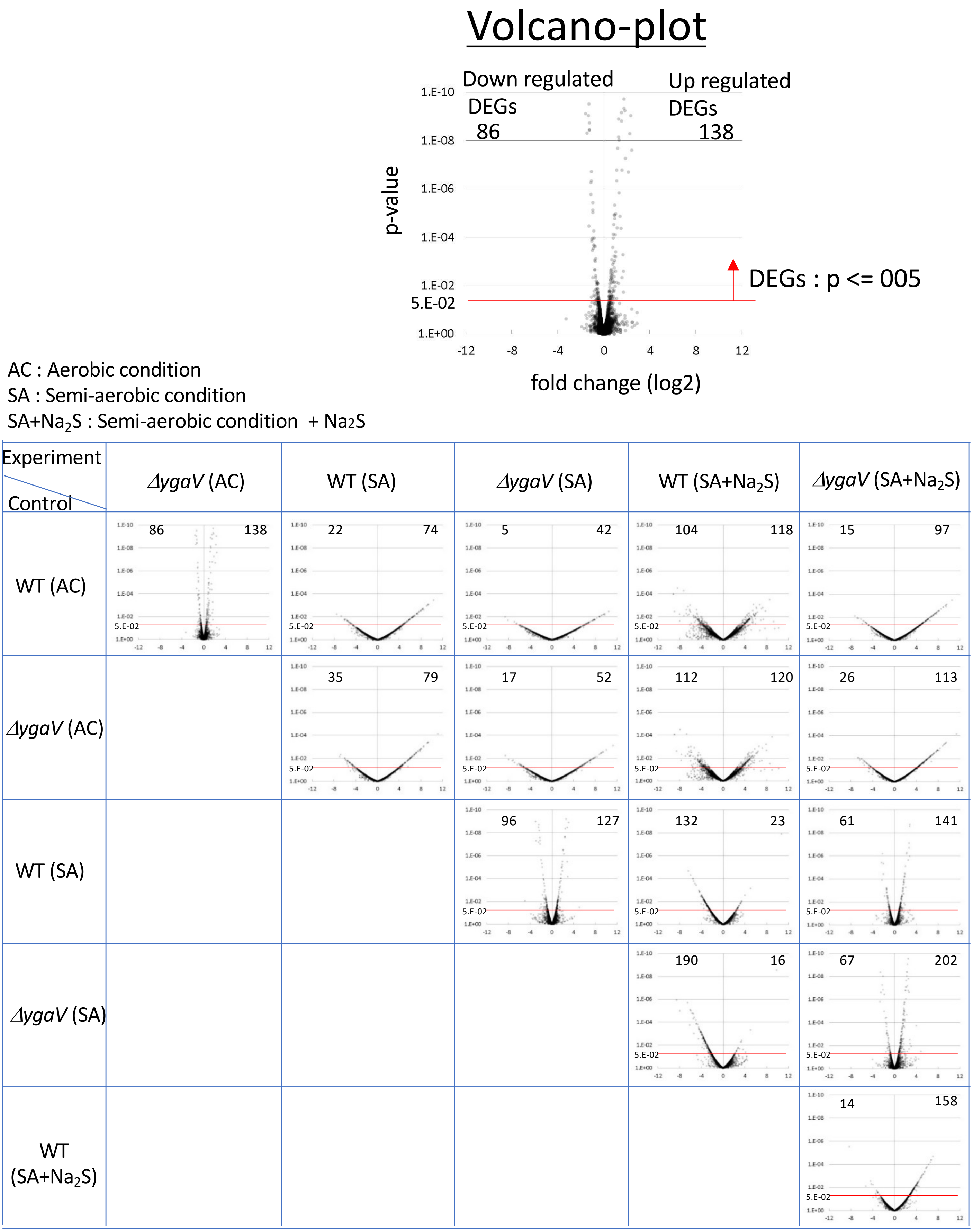

Supplemental Figure S2 Volcano plots for upregulated and downregulated transcripts in WT and *ΔygaV* mutant grown under aerobic (AC) and semi-aerobic (SA) conditions with (+Na_2S) or without 0.2 mM Na_2S.

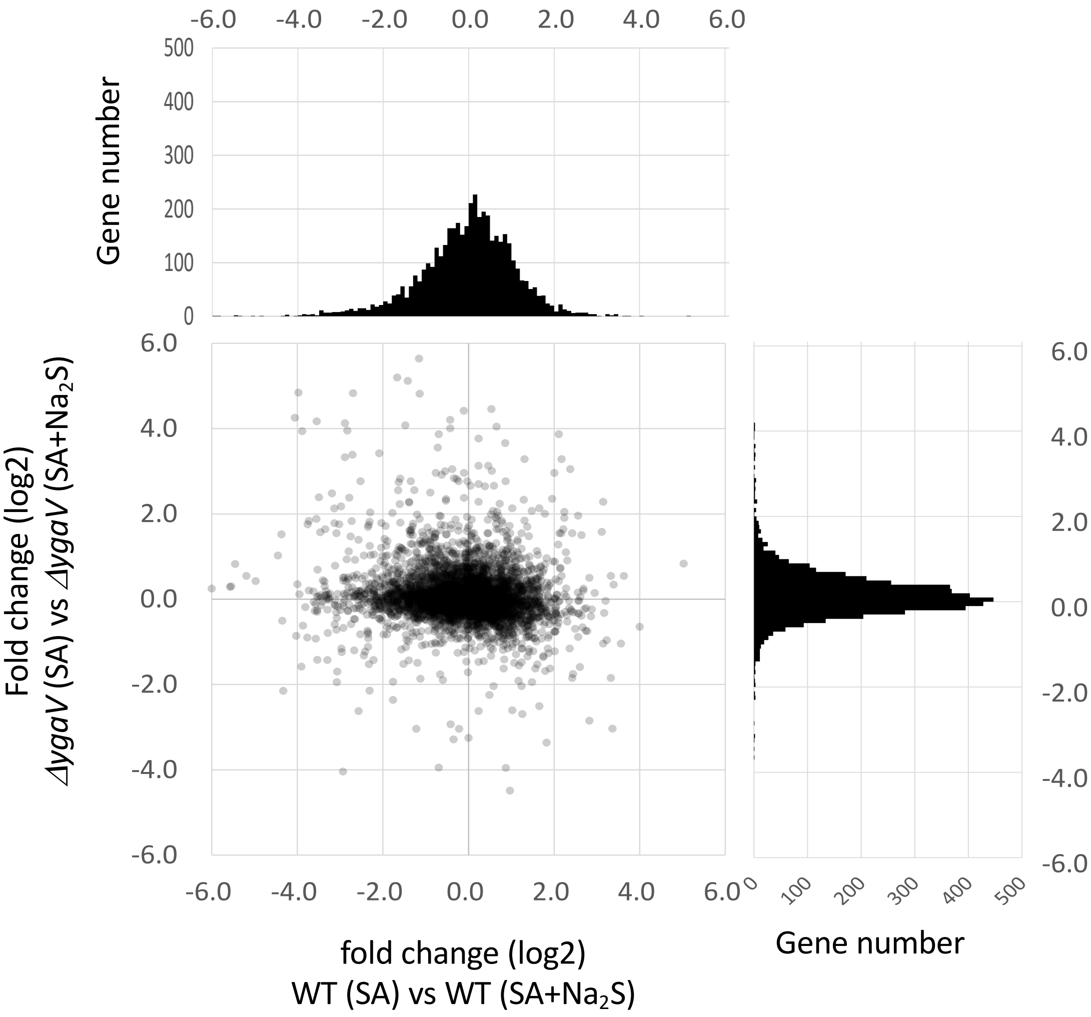

Supplemental Figure S3 Scatter plot depicting distributions of the expression changes between WT (SA) and WT (SA+Na_2S) (x-axis, top marginal histogram) against the expression changes between *ΔygaV* (SA) and *ΔygaV* (SA+Na_2S) (y-axis, right marginal histogram).

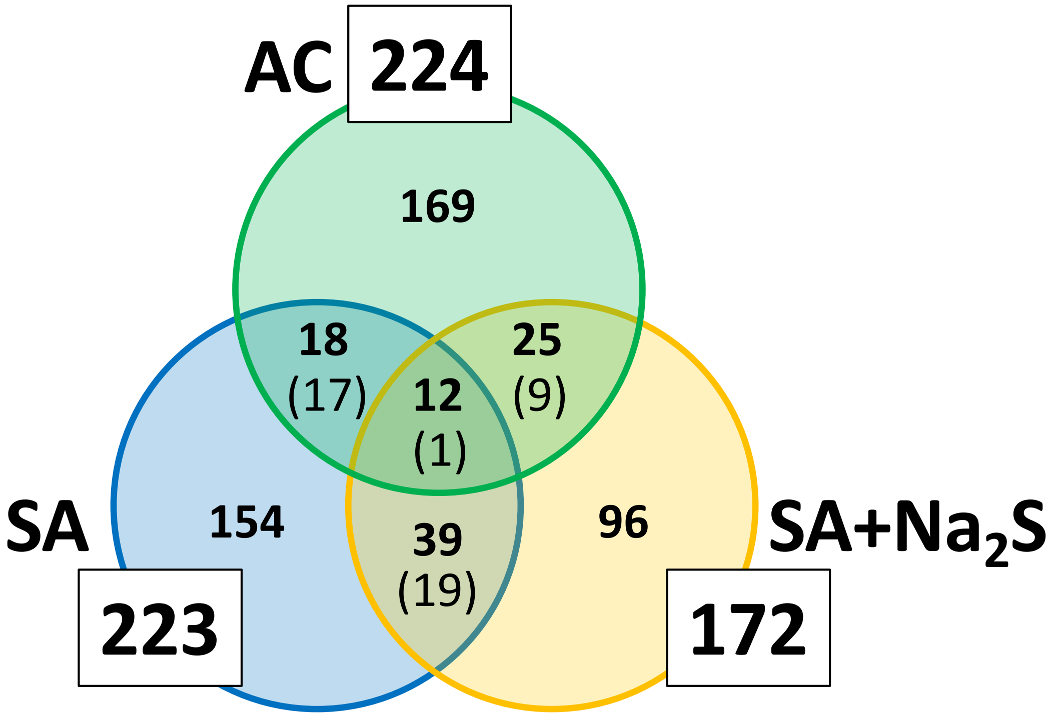

Supplemental Figure S4 Venn diagrams depict the overlap between genes differentially expressed in the *ΔygaV* mutant compared to WT, grown under aerobic (AC) and
semi-aerobic (SA) conditions, with (+Na_2S) or without 0.2 mM Na_2S. The numbers in brackets indicate the number of genes with concordant increased or decreased expression in the two comparisons.
